# Understanding the Importance of Fatherhood among Men Living with HIV in Ontario

**DOI:** 10.1177/23259582211016133

**Published:** 2021-05-18

**Authors:** Luke Thomas Kyne, Mark H. Yudin, Tsegaye Bekele, Mona Loutfy, Sean B. Rourke, James Watson, Joseph Nguemo Djiometio, Tony Antoniou, Jason Globerman, Adam McGee, V. Logan Kennedy

**Affiliations:** 1 Women’s College Research Institute, Women’s College Hospital, Toronto, Ontario, Canada; 2 Department of Obstetrics and Gynecology, University of Toronto, Toronto, Ontario, Canada; 3 10071 St. Michael’s Hospital, Toronto, Ontario, Canada; 4 Li Ka Shing Knowledge Institute, Toronto, Ontario, Canada; 5 269770 Ontario HIV Treatment Network (OHTN), Toronto, Ontario, Canada; 6 Department of Medicine, University of Toronto, Toronto, Ontario, Canada; 7 Department of Psychiatry, University of Toronto, Toronto, Ontario, Canada; 8 Centre for Urban Health Solutions, Toronto, Ontario, Canada; 9 Daphne Cockwell School of Nursing, Ryerson University, Toronto, Ontario, Canada; 10 Department of Family and Community Medicine, University of Toronto, Toronto, Ontario, Canada

**Keywords:** community-based research, fatherhood, men

## Abstract

While pregnancy and motherhood have become paramount clinical issues for women living with HIV, parenting has received less attention among men living with HIV (MLWH). We conducted a secondary analysis of a cross-sectional study assessing fertility desires and intentions of MLWH using a 5-point Likert scale based on the question: “Being a father is important to me”. Logistic regression models were fit to calculate unadjusted and adjusted odds ratios (ORs) and confidence intervals (CIs) for significant correlates. Of the 276 respondents, 118 were heterosexual, 158 were gay, bisexual, 2-spirit, or queer (GBTQ), 55% had never parented before, and 65% wanted to parent. 191 (69%) respondents agreed that fatherhood was important to them. In unadjusted analyses, heterosexuality (OR 1.52; 95% CI 1.15 to 2.03), African/Caribbean/Black ethnicity (OR 1.57; 95% CI 1.12 to 2.19), African/Caribbean birthplace (OR 1.48; 95% CI 1.06 to 2.05), and history of parenting (OR 1.60; 95% CI 1.10 to 2.39) were significantly (p < 0.05) associated with importance of fatherhood. However, none of these variables were significant in adjusted analyses. From the unadjusted model, factors such as sexual orientation, ethnicity, and current parenthood may influence how MLWH value fatherhood, suggesting HIV and fatherhood is complex and must be explored further.

## Introduction

It is undeniable that the dominant narrative on reproductive health issues—including parenthood in the context of HIV and reproductive rights and decision-making—has prioritized women.^1^ In fact, as early as 1984, literature emerged reporting on the clinical manifestations of acquired immune deficiency syndrome (AIDS) in pregnancy.^2^ What quickly followed throughout the 1990s was an increased focus on contraception, pregnancy, breastfeeding, and parenting for women living with HIV (WLWH) in low-, middle-, and high-income countries. More recently, focus has shifted away from solely biomedical concerns of pregnancy and motherhood towards incorporating psychosocial and cultural issues affecting WLWH.^3-5^ Factors like the ability to access contraception, reduce vertical transmission, and prenatal HIV testing have been highlighted alongside the importance, challenges, stressors, and triumphs of motherhood among WLWH.^6,7^ This discrepancy between WLWH and their male counterparts highlights a universal gender gap within the study of fertility, reproductive health, and parenthood well beyond HIV as women and girls continue to carry the global burden of reproductive health.

**Supplementary Questions**

**What Do We Already Know about This Topic?**

The importance that women living with HIV place on motherhood is already well known, while it is understood that experiences of fatherhood can help normalize and give meaning to life for men living with HIV (MLWH).

**How Does Your Research Contribute to The Field?**

This research contributes to the field by highlighting the importance MLWH place on fatherhood, which may correlate with demographic factors like sexual orientation, race, and history of parenting.

**What Are Your Research’s Implications Toward Theory, Practice, or Policy?**

The results of this study have implications for practice which suggest that clinicians and service providers should explore parenting desires in greater depth with MLWH.

Focusing on fathers in the context of the family unit is as relevant as motherhood in many regards. Indeed, an emerging body of literature has taken shape throughout the 2000s, specifically exploring the reproductive health and rights of men living with HIV (MLWH). While it remains a more limited body of literature, it does shed light on the important intersection of reproductive health and HIV among MLWH. Like women, many MLWH are undeterred from wanting and/or actually intending to become a parent. Studies have even reported that experiences of fatherhood help men move on from a positive diagnosis and normalize life with HIV.^1,8,9^ Additionally, it has been shown that the majority of MLWH feel raising children gives meaning to life and something to live for, while views on fathering have changed alongside treatment options.^10-12^ Increased parental involvement may also positively impact the lives of children with fathers living with HIV, resulting in improved grades and self-esteem.^13^ These findings cumulatively suggest that fatherhood may be an essential component in the lives of many MLWH.

However, the above findings remain limited in their scope and relevance to certain contexts, such as Canada. This is because the topic of fertility, reproductive health, and fatherhood in MLWH has garnered much greater attention in low- and middle-income countries with high HIV prevalence. In many of these settings, the social imperatives related to fatherhood are contextually derived and have limitations in their generalizability.^14-16^ Additionally, the majority of relevant literature on these topics was completed prior to the publication of landmark studies, namely HPTN052^17^ and PARTNERS.^18^ The resulting endorsement of U=U has changed the landscape of HIV forever, opening interest in and opportunities for pursuing parenthood that were unimaginable even a decade ago. Additionally, most of the existing literature has focused on either heterosexual men or men who have sex with men, recruited relatively small samples, and used limited methodological approaches.

The healthcare system also appears to have been less focused on MLWH with respect to fatherhood. This includes reduced access to testing and treatment information, neglectful health policies, and limited discussion about the reproductive intentions, decisions, and rights for men with HIV.^8^ As such, based on a strong desire expressed by people living with HIV in Ontario and the agencies that serve them, we undertook a study to explore various aspects of fertility and fatherhood among MLWH in Ontario. The fertility desires, intentions, and actions of study participants were assessed as the primary objective of the study and have been reported in detail elsewhere.^19^ To summarize, the study found that—similar to WLWH—a considerable proportion of participants desired children in the future (180/276: 89 heterosexual; 91 GBTQ). However, fertility intentions were lower (44%) than seen in a similar cohort of WLWH (57%) in Ontario.^3^

The current study presents a secondary analysis of the Ontario HIV Men’s Fertility Study data to elucidate the correlates influencing the value that MLWH place on fatherhood. Undertaking this analysis was deemed of particular importance given previous findings that suggest higher fertility rates among MLWH in pro-fatherhood societies.^9-11^ By identifying what characteristics correlate with pro-fatherhood ideologies among MLWH in Ontario, we aim to provide clinically relevant insight into what MLWH might be most likely to pursue fatherhood, thus increasing their need for comprehensive reproductive health counselling. We hypothesized that sexual orientation, ethnicity, and history of parenting would exhibit a significant correlation with the importance of fatherhood for MLWH. Closing the divide in our understanding of fatherhood and HIV is critical for the pursuit of equitable, effective, and comprehensive HIV care that could greatly benefit men and their current or future families.^20^ Explicating the sociodemographic considerations related to fatherhood are required to develop effective, community-specific health policies and practice to optimize the support of diverse MLWH.

## Methods

### Design, Population, and Survey Instrument

We performed a secondary analysis of a cross-sectional study assessing the fertility desires and intentions of MLWH from across Ontario, Canada. Participation in the study involved a single peer interviewer-administered survey. Peer interviewers were men and women living with HIV who had extensive experience in community-based research. The survey instrument was based on questions from an analogous survey investigating the fertility desires and intentions of WLWH^3^ and another survey assessing the reproductive views of MLWH in London, England.^10^ The survey development process, details of the survey contents, and the validation process have been reported elsewhere.^19,21^

Efforts were made to stratify participant enrolment to align regional distribution. To best match the sexual, ethnic, and geographical distribution of MLWH in Ontario, non-random, purposive sampling was used in participant recruitment. Participants were chosen based on the following inclusion criteria: (1) living with HIV; (2) 18 years of age or older; (3) identifying as male; (4) living in Ontario, Canada.

### Ethical Approval and Informed Consent

Primary research ethics approval was received from the University of Toronto’s Research Ethics Board (Reference # 33174). All participants provided written, informed consent indicating their agreement to complete the peer administered survey.

### Statistical Analysis

The importance of fatherhood for MLWH was the primary outcome of interest, based on the question: “Being a father is important to me”. Participants answered this question on a 5-point Likert scale ranging from “Strongly agree” to “Strongly disagree”. For the current analyses, the variable was dichotomized with men who answered “Strongly agree” or “Agree” considered to value the importance of fatherhood, whereas those who answered “Neither agree nor disagree”, “Disagree”, or “Strongly disagree” were considered to feel that fatherhood was not important to them. In this work, the term “fatherhood” was not specifically defined, and instead left up to the interpretation of participants.

All statistical analyses were completed using SAS Version 9.4 (SAS Institute, Cary, North Carolina, USA). Sample characteristics were summarized using median and inter-quartile range (IQR) for continuous variables and frequencies/percentages for categorical variables. Bivariable and multivariable logistic regression models were used to identify significant correlates of the importance of fatherhood. Sexual orientation, ethnicity, and history of parenting were a priori variables of interest. Other variables assessed included age, region in Ontario, birthplace, religion, education, employment status, income, relationship status, and HIV factors (e.g., years since HIV diagnosis, antiretroviral treatment, CD4 count, viral load, contraceptive use, coinfections). Associations were expressed as unadjusted and adjusted odds ratios (ORs) with 95% confidence intervals (CIs).

Variables with p < 0.20 in the bivariable analyses were candidates for inclusion in the final multivariable logistic regression model. In some cases, collinearity was observed between 2 variables (e.g., ethnicity and place of birth); when detected, the variable that showed stronger association with the outcome was retained in the model.

## Results

### Participant Demographics

A sample of 276 MLWH (118 heterosexual; 158 GBTQ) was recruited from 10 locations across Ontario, Canada (Table 1). The focus of this paper is specifically on understanding the importance of fatherhood within this sample of MLWH. There were 152 (43 heterosexual; 109 GBTQ) respondents that had never parented children, while 115 (71 heterosexual; 44 GBTQ) had parented at least one child. The demographics of the full study sample have been reported in detail elsewhere^19^ but are included in Table 1 for reference.

**Table 1.** Characteristics of Study Participants.

Characteristics	Whole sample (N = 276) n (%)
Sexual Orientation	
Heterosexual	118 (43%)
GBTQ	158 (57%)
Age in years	
18-40	86 (31%)
41-50	82 (30%)
51-60	89 (32%)
>60	19 (7%)
Ethnicity	
White	152 (55%)
African/Caribbean/Black	60 (22%)
Indigenous	29 (10%)
Asian/Hispanic/Middle Eastern	35 (13%)
Region in Ontario	
Greater Toronto Area (GTA)	136 (49%)
Eastern Ontario	50 (18%)
Southwestern Ontario	50 (18%)
Northern Ontario	40 (14%)
Birthplace	
Canada/USA	179 (65%)
Africa/Caribbean islands	56 (20%)
Asia/Europe/South America	41 (15%)
Religion	
Christian	150 (54%)
Atheist/Agnostic/none	77 (28%)
Indigenous/Buddhist/Muslim/Other	49 (18%)
Education level	
Less than HS completion	53 (19%)
HS completion or higher	223 (81%)
Employment status	
Working (FT/PT)	89 (32%)
School (FT/PT)	19 (7%)
On disability/social assistance	139 (50%)
Retired/other	29 (11%)
Household Income	
<$20,000	127 (46%)
$20,000 - $39,999	64 (23%)
≥$40,000	77 (28%)
Number of children parented (ever)	
0	152 (55%)
1	38 (14%)
2	36 (13%)
≥3	41 (15%)
Currently relationship	
Married/common-law/partnered	86 (31%)
Single, but in a romantic relationship	38 (14%)
Not in a romantic relationship	152 (55%)
Current partner’s HIV Status	
HIV-positive	51 (18%)
HIV-negative	56 (20%)
Don’t have a partner	164 (59%)
Had vaginal sex with a woman (ever)	
Yes	217 (79%)
No	59 (21%)
Had vaginal sex with a woman (last 12 months)	
Yes	72 (26%)
No	204 (74%)
Current contraceptive use	
Yes	42 (15%)
No/I don’t have female partner	234 (85%)
Years since HIV diagnosis	
0-5	49 (18%)
6-10	48 (17%)
11-15	54 (20%)
16-20	53 (19%)
>20	71 (26%)
HIV risk factor	
MSM/IDU	171 (62%)
Sex with a woman	50 (18%)
Other	20 (7%)
Don’t know/refused	35 (13%)
Current CD4 count	
≥200 cells/mm^3^	219 (79%)
Current viral load	
Undetectable	221 (80%)
Detectable/unknown	55 (20%)
Ever on ARV medication	
Yes	254 (92%)
No	16 (6%)
Currently on ARV medication	
Yes	241 (87%)
No	31 (11%)
Years since ARV initiation	
Median [IQR]	10 (4-15)
Sexually transmitted infections*	
Yes	72 (26%)
Want to have children in the future	
Yes	180 (65%)
No	96 (35%)
Intend to have children in the future	
Yes	121 (44%)
No	155 (56%)

### Importance of Fatherhood

Of the 276 participants, 191 (109 heterosexual; 89 GBTQ) respondents agreed with the primary question of interest, “Fatherhood is important to me”. Of the 191 participants in agreement, 125 (73 heterosexual; 52 GBTQ) expressed interest in future fatherhood, either agreeing or strongly agreeing with the statement “I would like to be a father in the future”. Of the 118 heterosexual men, 102 said fatherhood was important to them (86%), while 89 out of the 158 GBTQ men said fatherhood was important (56%) (p < 0.0001).

### Correlates of the Importance of Fatherhood

Following bivariate logistic regression analysis, the following variables were associated with the primary “importance of fatherhood” question: heterosexual identity (OR 1.52; 95% CI 1.15 to 2.03), African/Caribbean/Black ethnicity (OR 1.57; 95% CI 1.12 to 2.19), African/Caribbean birthplace (OR 1.48; 95% CI 1.06 to 2.05), and history of parenting (OR 1.60; 95% CI 1.10 to 2.39). However, these associations were no longer apparent following multivariable logistic regression analysis (Table 2). Stratified analyses between heterosexual and GBTQ men also resulted in no significant correlates.

**Table 2.** Bivariable and Multivariable Regression Models Predicting Importance of Fatherhood** among Men Living with HIV (n = 276).

Predictor variable	Bivariable regression			Multivariable regression		
	OR	(95% CI)	p	OR	(95% CI)	p
Age (Years)	1.00	(0.98, 1.01)	0.446			
Sexual orientation						
Heterosexual	1.52	(1.152.03)	**0.004**	1.24	(0.87, 1.75)	0.235
Gay, Bisexual, 2-Spirit, or Queer (ref)						
Ethnicity						
African/Caribbean/Black	1.57	(1.12, 2.19)	**0.008**	1.40	(0.96, 2.04)	0.079
Asian/Hispanic/Middle Eastern	1.15	(0.73, 1.80)	0.542	1.14	(0.68, 1.88)	0.623
Indigenous	1.21	(0.76, 1.95)	0.422	1.29	(0.80, 2.08)	0.295
White (ref)	1.00					
Region of Ontario						
Toronto	0.99	(0.75, 1.32)	0.947			
Other (ref)	1.00					
Birthplace						
Africa/Caribbean islands	1.48	(1.06, 2.05)	**0.020**			
Asia/Europe/South America	1.05	0.69, 1.59)	0.832			
Canada/USA (ref)	1.00					
Religion						
Atheist/Agnostic/None	0.93	(0.67, 1.30)	0.677			
Indigenous/Buddhist/Muslim/Other	1.01	(0.69, 1.48))	0.956			
Christian (ref)	1.00					
Relationship status						
Married/common-law/living with a partner	1.14	(0.84, 1.56)	0.407			
Single, but in a romantic relationship	1.03	(0.67, 1.59)	0.882			
Not in a romantic relationship (ref)	1.00					
Level of education						
HS completion or higher	0.99	(0.69, 1.42)	0.972			
Less than HS completion (ref)	1.00					
Work/school						
Working (FT/PT)	1.00	(0.59, 1.69)	0.989			
On disability/social assistance/student	1.14	(0.70, 1.88)	0.600			
Retired/other (ref)	1.00					
Annual household income						
≥40K	0.72	(0.50, 1.04)	0.080	0.86	(0.58, 1.28)	0.454
20K-39K	1.00	(0.71, 1.42)	0.988	1.08	(0.75, 1.55)	0.695
<20 K (ref)	1.00					
In a romantic relationship						
Yes	1.12	(0.85, 1.49)	0.418			
No (ref)	1.00					
Partner’s HIV status						
HIV-negative	1.13	(0.80, 1.59)	0.492			
HIV-positive/unknown/no partner (ref)	1.00					
Current contraceptive use						
Yes	1.61	(1.12, 2.30)	**0.010**			
No	1.64	(1.11, 2.41)	**0.012**			
No female partner (ref)	1.00					
Years since HIV diagnosis						
≤ 5	0.96	(0.63, 1.48)	0.858			
6-10	0.90	(0.58, 1.39)	0.630			
11-15	0.92	(0.61, 1.41)	0.707			
16-20	0.86	(0.56, 1.33)	0.506			
>20 (ref)	1.00					
Currently on ART						
Yes	0.93	(0.60, 1.43)	0.732			
No (ref)	1.00					
Current CD4 count (cells/mm^3^)						
≥200	0.93	(0.66, 1.31)	0.667			
<200/unknown (ref)	1.00					
Viral load						
Undetectable	0.94	(0.67, 1.34)	0.745			
Detectable/unknown (ref)	1.00					
Number of children parented (Ever)						
≥3	1.62	(1.10, 2.39)	**0.014**	1.37	(0.89, 2.12)	0.155
2	1.45	(0.95, 2.21)	0.086	1.34	(0.86, 2.06)	0.194
1	1.56	(1.04, 2.34)	**0.030**	1.52	(0.99, 2.32)	0.055
0 (ref)	1.00					

**Note:** Backward elimination method was used to identify predictor variables significantly associated with intention to have children.

**Note: “Agree” or “strongly agree” to importance of fatherhood question (“Being a father is important to me”).

**Bold** values represent variables with p < 0.20 which were candidates for inclusion in the multivariable logistic regression model.

## Discussion

In this study exploring the sociodemographic correlates of the importance of becoming a father among 276 MLWH, 69% of respondents stated that being a father was important to them, which included participants who had never parented children before and those who had parented at least one child previously. This was in alignment with other literature describing the importance all men place on fatherhood,^22-24^ as well as a 2018 publication on the values and ideals that heterosexual MLWH possess regarding fatherhood.^12^ Not surprisingly, advances related to cART, life expectancy, and messages such as U=U appear to have contributed to an increase in positive ideologies related to fatherhood among MLWH, as research from the early 2000s found slightly lower rates of pro-fatherhood ideals.^8,10^

An important contribution to the literature made by this study is the exploration of the impact of sexual orientation on the importance of fatherhood among a cohort of MLWH. The current study’s findings may imply that fatherhood is of greater importance to heterosexual MLWH than MLWH who identify as GBTQ. This finding appears consistent with the other literature related to fatherhood and GBTQ men. Specifically, previous literature has shown gay men are less likely to express a desire for parenthood than heterosexual men, and that gay men who wanted to become fathers were less likely to publicly voice their intentions to do so.^25^ Our finding might reflect heterosexual norms associated with fatherhood, which could influence the importance GBTQ men place on fatherhood compared to their heterosexual counterparts; or at least their willingness to report this importance. Our results and the increasing attention on the parenting rights of people within the LGBTQ+ community highlight the ongoing critical need to normalize the concept of fatherhood for all MLWH, so that potential fathers feel comfortable exploring and disclosing their desires, values, and intentions. It is of note that in our adjusted analysis, sexual orientation was not significantly associated with importance of fatherhood. This could be due to regional factors or other confounding variables, which may be necessary to explore before drawing conclusions between the relationship of sexual orientation and the importance of parenthood.

Our study also provides novel understanding of the role ethnicity plays in predicting pro-fatherhood ideals among a diverse population of MLWH. Ethnicity was found to be associated with importance of fatherhood in bivariate analysis, suggesting that ethnic and/or racial differences might alter the importance MLWH place on fatherhood. This aligns with previous work identifying differences in the importance that men from different ethnic backgrounds place on fatherhood.^1,9,15,16,26^ These findings also mirror research on motherhood and HIV, which has identified differences in the importance of motherhood across different ethnic groups.^4^ The collinearity between ethnicity and birthplace was expected due to their often-inextricable relationship and was considered in multivariable analysis. Again, in our adjusted analysis ethnicity was no longer significantly associated with importance of fatherhood. Nonetheless, further attention can be directed towards understanding the ethnic differences associated with fatherhood in the context of HIV.

A history of parenting also emerged as a significant variable related to the importance MLWH place on fatherhood. As these data suggest, it might make intuitive sense that men who were currently fathers would place a greater importance on fatherhood than those who were not. However, this may not properly reflect the views of all fathers, some of whom are less happy than their childless peers and may therefore place less of an importance on fatherhood.^22^ It is important to consider this alternative when interpreting our findings, as after adjusted analysis, current parenthood was revealed to be of no significance.

For clinicians and service providers, it is therefore necessary to consider that sexual orientation, ethnicity, and current parenthood may influence how MLWH value fatherhood. Other modelling suggests that fatherhood is more complex among MLWH and should be explored in greater depth during clinical encounters. Clinicians must continually avoid assumptions about who values fatherhood, and instead explore desires related to parenthood with all MLWH. Now more than ever, factors such as sex, gender, and age should not discriminate against pregnancy or parenthood. The authors of this paper were even asked to discuss this topic on a peer-run podcast, highlighting community interest surrounding HIV and fatherhood.^27^ By further exploring the concept of parenting with men in the context of HIV, optimal care can be provided.

## Limitations

Our study has some limitations. First, only a single question on fatherhood was addressed in our survey. Fatherhood was also not explicitly defined in this question, which may have resulted in individualized definitions of the term being utilized upon answering this survey item. However, the topic of HIV and fatherhood is expansive and can be further addressed using alternative research questions and methods. Second, this study consists of a convenience sample of MLWH in Ontario, and may therefore not reflect provincial, national, or global trends in fatherhood. The scope of the current study did not allow for an exploration of how other experiences of discrimination and inequity, such as poverty, housing instability, and food insecurity mediate the pro-fatherhood ideals of certain men living with HIV and their actual intent to parent. Research exploring these interactions would continue to improve our understanding of fatherhood and HIV. Also, this study only measured the self-reported importance that MLWH place on fatherhood; future research might include perspectives from partners and/or children.

## Conclusions

In this study, 69% of participating MLWH stated that being a father was important to them. Based on univariable analysis, we found that sexual orientation, ethnicity, birthplace, and history of parenting were all significant correlates regarding the importance of fatherhood within the target demographic. However, upon further multivariable analysis, none of these variables were significant. Although some relationships between variables and the primary question were elucidated, the topic of fatherhood and HIV is complex and must be explored in greater depth. Fatherhood can be a highly rewarding experience and may be transformative for men who become fathers. A diagnosis of HIV should not limit an individual’s ability to consider, discuss, and engage in parenthood. Clinicians and other service providers must continue to reduce biases where possible, and instead consider that fatherhood is an aspect of life which is important to the majority of MLWH. Only updates in health policy and clinical practice surrounding fatherhood will bring about widespread positive changes for MLWH. These changes should be actively promoted to increase the health and wellbeing of all MLWH. Additional investigations into contraception use and adoption opportunities in MLWH may provide a more comprehensive understanding of fatherhood in this population moving forward. By identifying and exploring correlates of the importance of fatherhood for MLWH, we hope to contribute towards improved health policy for those interested in parenting in the future.
